# High-Volume Transanal Surgery with CPH34 HV for the Treatment of III-IV Degree Haemorrhoids: Final Short-Term Results of an Italian Multicenter Clinical Study

**DOI:** 10.1155/2016/2906145

**Published:** 2016-02-21

**Authors:** Giuliano Reboa, Marco Gipponi, Maurizio Gallo, Giovanni Ciotta, Marco Tarantello, Angelo Caviglia, Antonio Pagliazzo, Luigi Masoni, Giuseppe Caldarelli, Fabio Gaj, Bruno Masci, Andrea Verdi

**Affiliations:** ^1^Coloproctology Unit, Casa di Cura San Camillo-Forte dei Marmi, Lucca, Italy; ^2^General Surgery and Breast Unit, IRCCS “San Martino-IST”, Genoa, Italy; ^3^Medical Oncology, IRCCS “San Martino-IST”, Genoa, Italy; ^4^General Surgery, Casa di Cura Triolo-Zancla e Villa Serena, Palermo, Italy; ^5^Coloproctology Unit, San Camillo Hospital, Rome, Italy; ^6^General Surgery, Villa Paideia Hospital, Rome, Italy; ^7^General Surgery, Celio Military Hospital, Rome, Italy; ^8^General Surgery, Policlinico Umberto I, Rome, Italy; ^9^General Surgery, San Carlo IDI Hospital, Rome, Italy; ^10^General Surgery, Fatebenefratelli Hospital, Rome, Italy

## Abstract

The clinical chart of 621 patients with III-IV haemorrhoids undergoing Stapled Hemorrhoidopexy (SH) with CPH34 HV in 2012–2014 was consecutively reviewed to assess its safety and efficacy after at least 12 months of follow-up. Mean volume of prolapsectomy was significantly higher (13.0 mL; SD, 1.4) in larger prolapse (9.3 mL; SD, 1.2) (*p* < 0.001). Residual or recurrent haemorrhoids occurred in 11 of 621 patients (1.8%) and in 12 of 581 patients (1.9%), respectively. Relapse was correlated with higher preoperative Constipation Scoring System (CSS) (*p* = 0.000),* Pescatori*'s degree (*p* = 0.000),* Goligher*'s grade (*p* = 0.003), prolapse exceeding half of the length of the Circular Anal Dilator (CAD) (*p* = 0.000), and higher volume of prolapsectomy (*p* = 0.000). At regression analysis, only the preoperative CSS,* Pescatori*'s degree,* Goligher*'s grade, and volume of resection were significantly predictive of relapse. A high level of satisfaction (VAS = 8.6; SD, 1.0) coupled with a reduction of 12-month CSS (Δ preoperative CSS/12 mo CSS = 3.4, SD, 2.0; *p* < 0.001) was observed. The wider prolapsectomy achievable with CPH34 HV determined an overall 3.7% relapse rate in patients with high prevalence of large internal rectal prolapse, coupled with high satisfaction index, significant reduction of CSS, and very low complication rates.

## 1. Introduction

**Figure 1:**
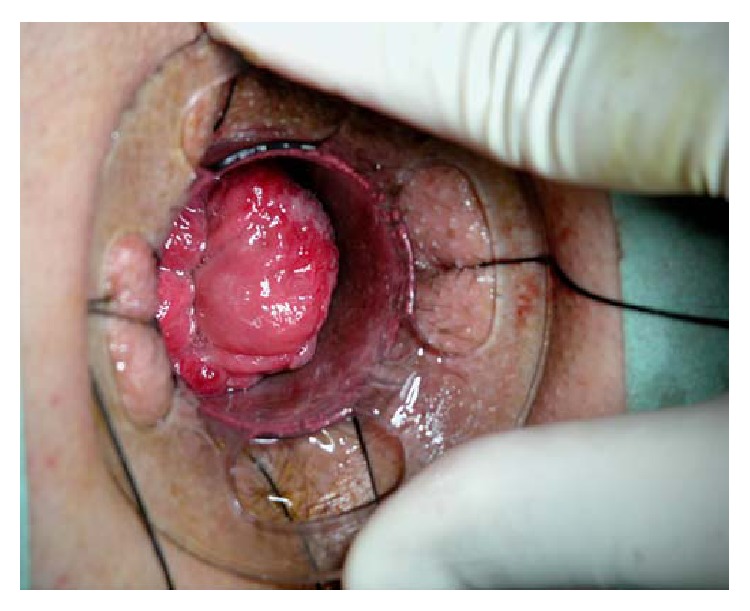
Rectal prolapse exceeding more than half of the length of the Circular Anal Dilator.

Stapled Haemorrhoidopexy (SH), by correcting the internal rectal prolapse associated with haemorrhoidal prolapse and interrupting the blood supply of the vascular cushions, has emerged as a more than convincing modality of treatment of patients with III-IV degree haemorrhoids. Actually, several clinical studies demonstrated that SH not only achieves less postoperative pain, superior functional recovery with earlier return to normal activities, and improved patient satisfaction with respect to Conventional Haemorrhoidectomy (CH) but it can also ameliorate the symptoms of obstructed defecation, frequently reported in these patients [1–8]. However, especially in patients with a large internal rectal prolapse, that is, a prolapse exceeding more than half of the length of the Circular Anal Dilator (CAD), a less than optimal prolapse resection can be performed by means of standard stapler devices with haemorrhoidal relapse up to 29.4% [9] (Figure 1). In order to accomplish a more satisfactory prolapse correction by resecting the excessive redundant tissue that determines the early haemorrhoidal recurrence, Stapled Transanal Rectal Resection (STARR) with PPH-01 or PPH-03 was proposed as a surgical option to overcome such technological limitations, with a significant reduction of residual and/or recurrent haemorrhoids [10–12].

**Figure 2:**
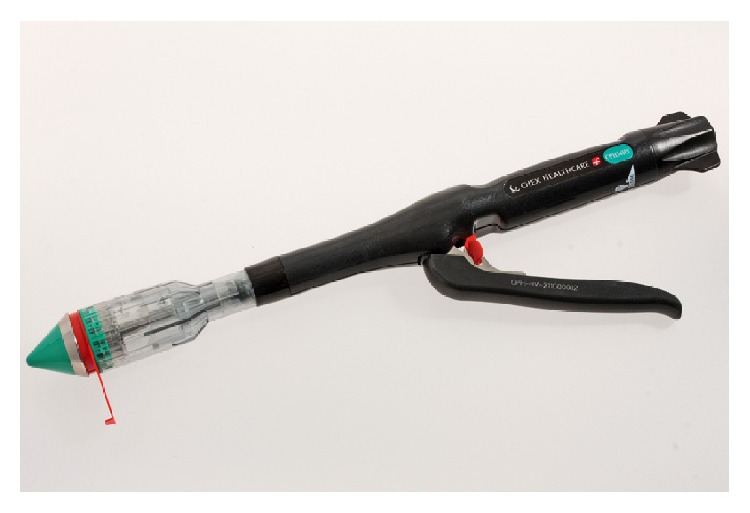
CPH34 HV.

Recently, a new device for high-volume (HV) transanal surgery, CVPH34 HV (Frankenman International Limited, Hong Kong), has been developed in order to guarantee a wider prolapse resection as compared to most of currently available staplers thanks to its high internal volume of the casing equal to 25 cm^3^ (Figure 2). Its safety as well as the higher volume of resection was experimentally confirmed, with a significant increase of both volume and weight of the tissue specimens as compared to PPH-03 (*p* = 0.0402 and *p* = 0.0375, resp.), the latter having approximately 35% less volume of resection [9].

On these grounds, a multicenter observational clinical study was undertaken in 430 patients with haemorrhoidal prolapse undergoing SH by means of CPH34 HV with the aim of assessing its safety and efficacy, with special care to the haemostatic properties of this HV stapler as well as the adequacy of prolapse resection. The interim data analysis indicated that the higher volume of prolapse resection achievable with CPH34 HV allowed reducing both the residual and recurrent haemorrhoidal prolapse, and this translated into a high index of patient satisfaction with an improvement of constipations scores [13]. After a complete 12-month follow-up, the efficacy and safety of SH by means of CPH34 HV were assessed in a wider sample of 621 patients with a high prevalence of large internal rectal prolapse associated with haemorrhoids.

## 2. Patients and Methods

The clinical charts of 621 patients with symptomatic III-degree or IV-degree haemorrhoids, 19 to 85 years of age, who underwent SH in the period 2012–2014, were consecutively reviewed. The following Surgical Centers participated to the study: (i) Coloproctology Unit, Casa di Cura San Camillo-Forte dei Marmi, Lucca (*n* = 348); (ii) General Surgery, Casa di Cura Triolo-Zancla, Palermo (*n* = 95); (iii) Colonproctology Unit, San Camillo Hospital, Rome (*n* = 38); (iv) General Surgery, Villa Paideia Hospital, Rome (*n* = 35); (v) General Surgery, Celio Military Hospital, Rome (*n* = 31); (vi) General Surgery, Policlinico Umberto I, Rome (*n* = 29); (vii) Surgical Division, San Carlo Nancy Hospital, Rome (*n* = 15); and (viii) Surgical Division, Fatebenefratelli, Rome, Italy (*n* = 30).

The study group consisted of 310 (49.9%) males and 311 (50.1%) females with a mean age of 51 years (SD, 12.7 years; range: 19–85 years). Inclusion and exclusion criteria, peri- and postoperative care and clinical parameters, follow-up data, and the surgical procedure that was performed in the same way by all the participants in this study have been already described [13]. Clinical follow-up consisted of outpatient visits that were scheduled at six and 12 months after the operation, as soon as the complete healing was achieved. Residual prolapse was defined as the reduction, without disappearance, of prolapsed tissue (haemorrhoids and/or rectal prolapse) within six months after the operation; recurrent disease was defined as the reappearance of prolapsed tissue after a symptom-free period of at least six months. Each patient gave his/her written informed consent and the study protocol was submitted to the Ethic Committee approval. All patients underwent clinical examination six and twelve months postoperatively in the outpatient clinic.

### 2.1. Statistical Analysis

A structured database was specifically developed for data collection; a descriptive analysis of all variables was performed. For numerical variables, the mean, standard deviation, and mean standard error were computed. Continuous variables were analyzed with a 2-tailed Student's *t*-test and binomial variables with chi-square analysis; a *p* value <0.05 was considered statistically significant. Univariate data analysis and logistic regression analysis were performed to assess those variables which were related to the occurrence of residual/recurrent haemorrhoidal prolapse.

## 3. Results

The clinical characteristics of patients are reported in Table 1. Constipation was a common finding in the study group as suggested by the mean constipation index (9.21; 2.8 SD) assessed by means of* Wexner*'s Constipation Scoring System [15]; patients who developed residual/recurrent haemorrhoidal prolapse had significantly higher constipation scores (mean = 11.95, SD = 1.89) as compared to patients without recurrence (mean = 9.1; SD = 2.7) (2-tailed Student's *t*-test; *p* value < 0.00001). Intraoperatively, 411 (66.2%) patients of 621 had an internal rectal prolapse exceeding more than half of the length of the CAD, while 210 (33.8%) had a rectal prolapse within half of the length of the CAD. A standard “Stapled Haemorrhoidopexy” was performed in the great majority of patients (*n* = 575; 92.6%), while the “Parachute” technique was used in 46 patients (7.4%). The mean operative time was 26.5 (SD, 6.6; range: 15–60) minutes. One technical failure of the device did occur (0.2%) without any untoward effect as for the operation. Only in a minority of patients haemostatic stitches were required to achieve complete haemostasis of the suture line, with a mean number of 1.4 stitches/patients (SD, 1.7; range: 0–8). Associated procedure was performed in 327 (52.7%) of patients, such as skin tags excision (*n* = 158; 25.4%), anal fissure diathermy (*n* = 121; 19.5%), condyloma excision (*n* = 8; 1.3%), and fistulotomy/fistulectomy (*n* = 3; 0.5%). The mean in-hospital stay was 1.5 days (SD, 1; range: 1–5); it was prolonged beyond one day in 23 patients (3.7%) due to mild bleeding or postoperative pain, representing the more frequent early postoperative complications (Table 2).

After stratification by the extent of the internal rectal prolapse, the mean volume of the doughnuts was significantly higher (13.0 mL; SD, 1.4) in the group of 390 patients with an internal rectal prolapse exceeding more than half of the length of the CAD compared to the group of 192 patients with smaller prolapse (9.3 mL; SD, 1.2) (*p* value < 0.001), while no significant difference was observed by type of operation (Table 3).

At 6-month and 12-month follow-up assessment, residual and recurrent haemorrhoidal prolapse occurred in 11 of 621 patients (1.8%) and in 12 of 581 patients (1.9%), respectively, all of them having originally a large internal rectal prolapse exceeding more than half of the length of the CAD (Tables 4 and 5). At univariate analysis, haemorrhoidal relapse was significantly related to higher preoperative CSS (*p* = 0.000),* Pescatori*'s degree of internal rectal prolapse (*p* = 0.000),* Goligher*'s grade of heamorrhoids (*p* = 0.003), prolapse exceeding half of the CAD (*p* = 0.000), and higher volume of prolapse resection (*p* = 0.000) (Table 6). At logistic regression analysis only the preoperative CSS (*p* < 0.004),* Pescatori*'s degree (*p* < 0.001),* Goligher*'s grade (*p* < 0.01), and volume of resection (*p* < 0.02) were significantly predictive of relapse.

A high index of patient satisfaction (Visual Analogue Scale = 8.6; SD, 1.0) coupled with a reduction of 12-month CSS as compared to the preoperative assessment (Δ preoperative CSS/12 mo CSS = 3.4, SD, 2.0; *p* < 0.001) was observed.

## 4. Discussion

SH is an innovative surgical treatment of haemorrhoids based on the pathophysiological concept of correcting the internal rectal prolapse thought to determine the sliding down of haemorrhoids from the anal canal, in order to “lifts” them back into their original anatomic site. This is accomplished by means of the circular excision of a variable volume of rectal wall, without any excision of the prolapsed haemorrhoids; this avoids any wound in a very sensitive area such as the anus and perianal skin thus determining less postoperative pain, superior functional recovery with earlier return to normal activities, and improved patient satisfaction [1–8].

However, the risk of residual/recurrent haemorrhoids is two- to three-fold higher after SH as compared to CH, with a rate up to 29.4% in patients with large internal rectal prolapse [7, 10–12]. For these reasons, STARR has been proposed as an alternative to SH in patients with large internal rectal prolapse, that is, a prolapse exceeding half of the longitudinal length of the CAD at the intraoperative assessment. Clinical studies have confirmed the significant reduction of residual/recurrent disease by means of the STARR procedure; noteworthy, the recurrence rate in the specific subset of patients with a large internal rectal prolapse was reduced to 1.9–5.9% [10–12]. This improvement is mainly related to the wider extent of prolapse resection amenable with the STARR procedure because neither the details of the operation* per se* nor the deeper rectal wall resection that is thought to be accomplished with STARR may justify these results. As a matter of fact, the historical distinction between SH and STARR based on the thickness of the doughnuts (mucosal* versus* full-thickness rectal wall resection) has lost much of its clinical relevance as smooth muscle fibres can be found in 4% to 97% of excised mucosal rings after SH [16]. However, the STARR procedure for haemorrhoids should be very selectively proposed due to the costs of the devices as well as the higher risk of postoperative complications.

So, what we actually would need is not a double prolapsectomy but the possibility to use new HV staplers, such as the CPH34 HV, in order to guarantee higher volumes of prolapse resection in order to correct more properly the internal rectal prolapse. Following experimental testing that confirmed both higher volumes of resection and less anastomotic bleeding, a multicenter clinical study was started in order to verify the safety and the efficacy of CPH34 HV, especially in terms of postoperative bleeding and extent of prolapse resection [9, 13]. Preliminary results indicated that the higher volume of prolapse resection achievable with CPH34 HV allowed to reduce both residual and recurrent haemorrhoidal prolapse, and this translated into a high index of patient satisfaction with an improvement of constipations scores [13].

Now, after complete a 12-month follow-up was performed in a wider sample of patients with a high prevalence (66.2%) of large internal rectal prolapse associated with haemorrhoids, the efficacy of SH was confirmed because the wider prolapsectomy achievable with CPH34 HV allowed reducing to 3.7% the overall rate of haemorrhoidal relapse coupled with high satisfaction index and a significant improvement of constipation scores (*p* < 0.001). This implies a five- to six-fold decrease of postoperative haemorrhoidal relapse as compared to previous clinical experiences with SH performed in patients with a large internal rectal prolapse (25–29.4%) [7, 10]. Moreover, the 12-month haemorrhoidal relapse rate that was observed in this clinical experience was lower as compared to the historical outcome of patients undergoing CH, that is, approximately 5%, thus solving one of the last drawbacks of SH even in patients with a high prevalence of medium-large internal rectal prolapse [17]. Similarly, a rather high rate of haemorrhoidal recurrence, up to 25.4%, is reported especially in patients with grade IV haemorrhoids undergoing Trananal Haemorrhoidal Dearterialisation (THD) and Rectoanal Repair (RAR), with 19% of patients requiring further medical or surgical treatment [18–20].

These favourable clinical findings of HV transanal surgery are well explained by the high mean volume of the doughnuts (11.8 mL) that was almost double as compared to those retrieved after SH performed with conventional devices (6-7 mL) [21]. It is worth noting that specimen volumes up to 18 mL were recovered in patients with a large rectal prolapse, thus meaning that approximately 72% of the internal volume (25 cm^3^) of the stapler casing of the CPH34 HV is “really” available for prolapse resection while with the traditional PPH03 (17.4 cm^3^) no more than 40% of the casing is available for prolapse resection [11].

Notably, the univariate analysis suggested that patients with haemorrhoidal relapse had significantly higher CSS scores,* Pescatori*'s degree of internal rectal prolapse,* Goligher*'s degree of haemorrhoids, and a wider extent of prolapse both at the intraoperative and at resection specimen assessment. It is worth noting that the reduction of 6-month and 12-month CSS scores as compared to preoperative values was not significantly different in patients with and without haemorrhoidal relapse thus suggesting that SH determined a similar improvement of outlet obstruction symptoms, although patients who relapsed had significantly higher preoperative CSS scores (*p* = 0.000) related to the larger internal rectal prolapse that precluded its satisfactory correction even with a HV device. Overall, this means that there is a subset of patients with haemorrhoidal disease and an associated large internal rectal prolapse that shares the common clinical features of Obstructed Defecation Syndrome, so that they might benefit of an even wider prolapsectomy that is currently achievable with a STARR Mono-Stapler performed by means of a CPH36 SMS [22].

As for postoperative complications, both intraoperative and early postoperative bleeding occurred in a minority of patients because only very few haemostatic stitches (1.4/patient) were required for the intraoperative control of anastomotic bleeding: it is worth noting that a minor postoperative bleeding, well managed with conservative measures, occurred in 23 patients (3.7%) only, while this is usually reported in up to 30% of patients, thus confirming the more than satisfactory haemostatic properties of CPH34 HV [5].

Moreover, another frequent early postoperative complaint in patients undergoing SH is represented by spontaneous and/or postdefecation anal pain. In our experience, this symptom was reported only in 7.2% of patients and, when occurring, it was usually well controlled with mild analgesics with no need of hospital readmission. Also faecal urgency occurred in a minority of patients (4.7%) but was almost completely solved at 12-month follow-up (0.8%). These complications are mainly related to the surgeon's learning curve and, also, to a frequently neglected detail of the operation, namely, the “*Controlled Digital Stretching*” prior to the insertion of the operating proctoscope [23–25]. As a matter of fact, this preliminary manoeuvre aids in reducing the high preoperative anal pressure which is frequently reported in patients with haemorrhoidal disease that may impair the ability to satisfactorily evacuate the rectum in the early postoperative period [26]. However, it should be carefully avoided in elderly patients, in subjects with a weak sphincter as well as in multiparous female patients or with previous surgery that weakened the anal sphincters.

## 5. Conclusions

This multicentric clinical study in patients undergoing SH by means of CPH34 HV for haemorrhoids, with a high prevalence of associated large internal rectal prolapse, suggests at short-term follow-up that the wider prolapsectomy achievable with CPH34 HV allowed a reduction to 3.7% the overall rate of haemorrhoidal relapse coupled with high satisfaction index, significant reduction of CSS, and very low complication rates mostly related to bleeding, fecal urgency, and postdefecation anal pain. These findings require long-term follow-up before drawing definitive conclusions. Moreover, there may be a subset of patients with haemorrhoidal disease sharing features of Obstructed Defecation Syndrome who might benefit from the even wider prolapsectomy that is currently achievable with a STARR Mono-Stapler performed by means of a CPH36 SMS.

## Figures and Tables

**Table 1:** Clinical characteristics of patients (*N* = 621).

		*N*.	%
Age, yrs			
Mean (SD)	51 (12.7)		
Range	19–85		
Sex			
Male		310	49.9
Female, *n*. (%)		311	50.1
Specific symptoms			
Pain score (VAS: 0–10)			
Mean (SD)	4.0 (1.7)		
Range	0–10		
Bleeding, *n*. (%)		606	97.6
Haemorrhoidal prolapse, *n*. (%)		619	99.7
Constipation, *n*. (%)		488	78.6
Soiling, *n*. (%)		86	13.8
Diarrhoea, *n*. (%)		6	1.0
*Pescatori*'s Degree of Rectal Prolapse^*∗*^			
I		5	0.8
II		513	82.6
III		103	16.6
Goligher's classification			
III		205	33.0
IV		416	67.0
*Wexner*'s Constipation Scoring System^*∗∗*^			
Mean (SD)	9.21 (2.8)		
Range	1–15		
Previous anorectal surgery		75	12.1

SD: standard deviation. ^*∗*^ *Pescatori*'s Degree of Rectal Prolapse: I degree, prolapse detectable below the anorectal ring on straining; II degree, prolapse reaching the dentate line; III degree, prolapse reaching the anal verge [14]. ^*∗∗*^ *Wexner*'s Constipation Scoring System: minimum score, 0; maximum score, 30 [15].

**Table 2:** Intra- and early postoperative findings (*N* = 621 patients).

		*N*.	%
Operative time, minutes			
Mean (SD)	26.5 (6.6)		
Range	15–60		
Prolapse exceeding more than half of the length CAD			
No		210	33.8
Yes		411	66.2
Type of prolapsectomy			
Standard “Stapled Haemorrhoidopexy”		575	92.6
“Parachute” technique		46	7.4
Haemostatic stitches, *n*.			
Mean (SD)	1.4 (1.7)		
Range	0–8		
Technical failures of the device		1	0.2
Associate procedures, *n*. (%)		327	52.7
Skin tags excision		158	25.4
Anal fissure		121	19.5
Condiloma		8	1.3
Fistulotomy/fistulectomy		3	0.5
Miscellaneous		37	5.8
Hospital stay, days			
Mean (SD)	1.5 (1.0)		
Range	1–5		
Early postoperative complications		139	22.4
Anal pain (spontaneous/postdefecation)		45	7.2
Bleeding		23	3.7
Acute urinary retention		26	4.2
Urgency		29	4.7
Thrombosed haemorrhoids		4	0.6
Others		12	1.9
Reoperation (within 30 days)		5	0.8

SD: standard deviation.

**Table 3:** Specimen measures available, stratified by type of prolapsectomy (standard “Stapled Haemorrhoidopexy” or “Parachute” technique) and extent of rectal prolapse.

	Mean (SD)	Range
Total patients (*N* = 582)		
Length, mm	82.8 (5.4)	69–96
Height, mm	37.6 (3.3)	25–45
Volume, mL	11.8 (2.2)	7–18
Standard “Stapled Haemorrhoidopexy” (*N* = 575)		
Length, mm	82.6 (5.3)	69–95
Height, mm	37.6 (3.3)	25–45
Volume, mL	11.7 (2.2)	7–18^*∗*^
“Parachute” technique (*N* = 46)		
Length, mm	86.8 (5.4)	76–96
Height, mm	38.3 (3.2)	32–45
Volume, mL	13.7 (2.5)	8–18^*∗*^
Prolapse more than half of CAD (*N* = 390)		
Length, mm	85.6 (3.8)	70–96
Height, mm	38.9 (2.6)	30–45
Volume, mL	13.0 (1.4)	7–18^*∗∗*^
Prolapse less than half of CAD (*N* = 192)		
Length, mm	77.2 (3.6)	69–91
Height, mm	35.0 (3.0)	25–45
Volume, mL	9.3 (1.2)	7–17^*∗∗*^

SD: standard deviation.

^*∗*^ *p* = 0.08.

^*∗∗*^ *p* < 0.001.

**Table 4:** Follow-up at six months in 621 patients.

		Δ	*p*	*N*	%
Residual disease (within six months)					
Spontaneous pain score (VAS: 0–10)					
Mean (SD)	0.1 (0.5)	3.9 (1.8)	<0.001		
Range	0–7				
Pain at defecation (VAS: 0–10)					
Mean (SD)	0.2 (0.6)				
Range	0–7				
Bleeding, *n*. (%)				10	1.6
Residual haemorrhoidal prolapse				11	1.8
Other symptoms/signs					
Urgency				27	4.3
Pruritus				7	1.1
Soiling				9	1.4
Incontinence				0	—
Anal stenosis				1	0.2
Anal fissure/abscess/fistula				1	0.2
Haemorrhoidal thrombosis				2	0.3
Residual skin tags				12	1.9
Patient satisfaction (VAS: 0–10)					
Mean (SD)	8.2 (1.2)				
Range	2–10				
Constipation Scoring System					
Mean (SD)	6.5 (2.4)	2.7 (1.8)	<0.001		
Range	0–13				

SD: standard deviation; Δ: absolute difference as compared to basal assessment.

**Table 5:** Follow-up at 12 months in 581 patients.

		Δ	*p*	*N*	%
Recurrent disease (after six months)					
Spontaneous pain score (VAS: 0–10)					
Mean (SD)	0.1 (0.6)	3.9 (1.8)	<0.001		
Range	1–3				
Pain at defecation (VAS: 0–10)					
Mean (SD)	1.2 (0.5)				
Range	1–3				
Bleeding, *n*. (%)				12	1.9
Recurrent haemorrhoidal prolapse				12	1.9
Other symptoms/signs					
Urgency				5	0.8
Pruritus				21	3.4
Soiling				18	2.9
Incontinence				0	—
Anal stenosis				1	0.2
Anal fissure/abscess/fistula				4	0.6
Haemorrhoidal thrombosis				2	0.3
Residual skin tags				1	0.2
Patient satisfaction (VAS: 0–10)					
Mean (SD)	8.6 (1.0)				
Range	4–10				
Constipation Scoring System					
Mean (SD)	5.8 (2.0)	3.4 (2.1)	<0.001		
Range	0–13				

SD: standard deviation; Δ: absolute difference as compared to basal assessment.

**Table 6:** Univariate analysis in patients with and without hemorrhoidal relapse.

Parameter	No haemorrhoidal relapse		Haemorrhoidal relapse		*χ* ^2^	*p*
	*N* (%)	Mean (SD)	*N* (%)	Mean (SD)		
CSS						
Preoperative		9.1 (2.8)		11.9 (1.9)		0.000
Δ (Preop/6 mo)		2.7 (1.8)		2.5 (1.1)		0.607
Δ (Preop/12 mo)		3.4 (2.1)		3.6 (1.5)		0.420
*Pescatori*'s degree of prolapse						
II	510 (85.3)		8 (34.8)		40.83	0.000
III	88 (14.7)		15 (65.2)			
*Goligher*'s classification						
III	204 (34.1)		1 (4.3)		8.87	0.003
IV	394 (65.9)		22 (95.7)			
Type of operation						
Standard	556 (93.0)		19 (82.6)		3.47	0.062
“Parachute” technique	42 (7.0)		4 (17.4)			
Extent of prolapse						
Within half of the CAD	210 (35.1)		0 (0.0)		12.20	0.000
Exceeding half of the CAD	388 (64.9)		23 (100.0)			
Volume of prolapse		11.73 (2.24)		13.54 (1.60)		0.000

CSS: score of the Constipation Scoring System; SD: standard deviation; *χ* ^2^: Chi-square; extent of the prolapse exceeding or not half of the CAD (intraoperative assessment); volume of the prolapse (operative specimen assessment).
